# Interleukin 6 Accelerates Mortality by Promoting the Progression of the Systemic Lupus Erythematosus-Like Disease of BXSB.*Yaa* Mice

**DOI:** 10.1371/journal.pone.0153059

**Published:** 2016-04-06

**Authors:** Shweta Jain, Giljun Park, Thomas J. Sproule, Gregory J. Christianson, Caroline M. Leeth, Hongsheng Wang, Derry C. Roopenian, Herbert C. Morse

**Affiliations:** 1 Virology and Cellular Immunology Section, Laboratory of Immunogenetics, National Institute of Allergy and Infectious Diseases, National Institutes of Health, Rockville, MD, United States of America; 2 The Jackson Laboratory, Bar Harbor, ME, United States of America; 3 Hematology Research Unit, University of Helsinki, Helsinki University Central Hospital Cancer Center, Biomedicum Helsinki 1, Haartmaninkatu, Helsinki, Finland; 4 Department of Animal and Poultry Sciences, College of Agriculture and Life Sciences, Virginia Tech, Blacksburg, VA, United States of America; INSERM-Université Paris-Sud, FRANCE

## Abstract

IL6 is a multifunctional cytokine that drives terminal B cell differentiation and secretion of immunoglobulins. IL6 also cooperates with IL21 to promote differentiation of CD4^+^ T follicular helper cells (T_FH_). Elevated serum levels of IL6 correlate with disease flares in patients with systemic lupus erythematosus (SLE). We previously reported that IL21 produced by T_FH_ plays a critical role in the development of the SLE-like disease of BXSB.*Yaa* mice. To examine the possible contributions of IL6 to disease, we compared disease parameters in IL6-deficient and IL6-competent BXSB.*Yaa* mice. We report that survival of IL6-deficient BXSB.*Yaa* mice was significantly prolonged in association with significant reductions in a variety of autoimmune manifestations. Moreover, B cells stimulated by co-engagement of TLR7 and B cell receptor (BCR) produced high levels of IL6 that was further augmented by stimulation with Type I interferon (IFN1). Importantly, the frequencies of T_FH_ and serum levels of IL21 were significantly reduced in IL6-deficient mice. These findings suggest that high-level production of IL6 by B cells induced by integrated signaling from the IFN1 receptor, TLR7 and BCR promotes the differentiation of IL21-secreting T_FH_ in a signaling sequence that drives the lethal autoimmune disease of BXSB.*Yaa* mice.

## Introduction

Systemic lupus erythematosus (SLE) is a multigenic autoimmune disorder characterized by the formation and accumulation of immune complexes that cause tissue damage. In humans, the disease is more prevalent in females than males. Approximately 40 genes have been identified as genetic susceptibility loci for the disease, revealing the complexity of inheritance that underlies the disorder [1]. Cardinal features of SLE include chronic B cell activation accompanied by the presence of antibodies to a range of self-antigens including anti-nuclear and anti-double stranded DNA antibodies (ANA and dsDNA, respectively). Studies of genetically-programmed mouse models of SLE have advanced our understandings of the mechanisms involved in disease pathogenesis. BXSB.*Yaa* mice develop a severe humoral autoimmune disease that shares many features with human SLE. Male mice of this strain carry the Y-linked autoimmune accelerator (*Yaa*) mutation due to the chromosomal translocation of a portion of the X to the Y chromosome resulting in a duplication of a number of genes including *Tlr7* [2, 3]. This results in male mice carrying two functional copies of *Tlr7* in contrast to BXSB females that express only one functional copy due to X inactivation and BXSB males carrying a wild-type Y-chromosome. The doubling of *Tlr7* expression in BXSB.*Yaa* males is sufficient to greatly accelerate the mild, late onset SLE-like autoimmune disease observed in females after 1 year of age to a much more acute disease with deaths averaging 6 months of age [4, 5]. TLR7 binds single stranded RNA (ssRNA) leading to activation of a signaling cascade that results in the production of Type I IFN (IFN1) and inflammatory cytokines including TNFα and IL6 [6–8]. In addition, disease flares in human SLE are often associated with heightened expression of IL6 as well as IFN1 that heightens B cell sensitivity to various stimuli.

Previous studies centering on CD4^+^ T follicular helper cells (T_FH_) have identified several additional major determinants of disease. First, IL21, a γ_c_ cytokine produced primarily by T_FH_ and extrafollicular T_FH_ in BXSB.*Yaa* mice, is an absolute requirement for disease [9, 10]. This conclusion is based on studies showing that mice deficient in expression of the IL21 receptor (IL21R) exhibited no signs of disease, thereby identifying IL21 as a non-redundant cytokine central to disease initiation and progression [9]. Second, disease progression in BXSB.*Yaa* mice is restricted by an axis governed by CD8^+^ T suppressor (Ts) cells and NK cells. These cell populations are also dependent on IL21 as shown by studies demonstrating that the appearance of multiple signs of disease and as well as mortality are greatly accelerated in BXSB.*Yaa* mice bearing null alleles of both *Cd8* and *Il15* [10] and that the regulatory activity of CD8 T cells depends on IL21 signaling [11]. Thus, the development of T_FH_ that secrete high levels of IL21 is a central component of the BXSB.*Yaa* disease.

The mechanisms that drive the expansion of T_FH_ and heightened expression of IL21 by T_FH_ in BXSB.*Yaa* mice have not been defined. IL6 is an attractive candidate as a promotor of these phenotypes and, more generally, of the pathogenesis of SLE. A number of studies in humans and mice have identified roles for IL6 in conjunction with IL21 in T_FH_ differentiation [12–16]. Recently, it was shown that activation of STAT1 by IL6 is critical to the transcriptional activation of *Bcl6* and *Cxcr5* in the early processes of T_FH_ differentiation with IL21 appearing to contribute to the later phases of the differentiation program [17]. In addition, the established roles of IL6 in late B cell development suggest it may contribute to the massive accumulations of extrafollicular plasmablasts and plasma cells identified in lymphoid tissues of BXSB.*Yaa* mice [10]. Possible contributions of IL6 to disease in human SLE and in mouse models of lupus were first suggested by the identification of elevated levels of IL6 levels in sera of patients with SLE [18–22]. Mouse models of SLE, such as MRL.*Fas*^*lpr*^ have been found to have increased serum levels of IL6 that progress with age [18, 19]. In addition, IL6 was shown to increase the B cell-mediated autoantibody production in NZB/W F1 mice [23]. Finally, IL6 has been identified as one of the major genetic risk factors for human SLE as determined by genome-wide association studies (GWAS) [24].

The present study was aimed at determining whether IL6 plays an important role in the development of the SLE-like disease of BXSB.*Yaa* mice. We observed that BXSB.*Yaa* mice had elevated serum levels of IL6 that correlated with the appearance and progression of disease findings, including the expansion of T_FH_. We also found that B cells, which exhibited an activated phenotype, were a prominent source of IL6. Remarkably, the survival of BXSB.*Yaa* mice homozygous for a null allele of *Il6* (*Il6*^*-/-*^) was greatly prolonged over that of WT mice, and major signs of disease in younger mice were markedly attenuated.

## Materials and Methods

### Mice

BXSB.*Yaa*/*MpJ* (JR740) mice from the Jackson Laboratory were housed at a NIAID or Jackson Laboratory animal facility. Male and female mice of this strain are designated as BXSB.*Yaa* and BXSB respectively. IL6-deficient BXSB.*Yaa* mice (BXSB.*YaaIl6*^*-/-*^ or BXSB.*Il6*^*+/-*^*)* were generated by crossing the IL6 knockout construct from B6.129S2-*Il6*^*-/-*^(B6.129S2-*Il6*^*tm1Kopf*^/J, JR2650) with BXSB.*Yaa/MpJ* mice for 11 generations. Mice were genotyped for the *Il6* knockout allele using oligonucleotide primer sequences TCCATCCAGTTGCCTTCTTGG (common), TTCTCATTTCCACGATTTCCCA (wild type reverse) and CGGAGAACCTGCGTGCAAT (mutant reverse). In some experiments, BXSB.*B6Y* consomic controls (BXSB.B6-*Yaa*^+^/MobJDcr) were used in which the Y chromosome from C57BL/6J was transferred onto BXSB, thereby replacing the BXSB *Yaa* chromosome. All mice used in the study were maintained under specific pathogen free conditions and were fed with standard feed and water *ad libitum*.

### Ethics Statement

Animal Ethics Committees of NIAID, NIH and the Jackson Laboratory approved all experiments. The experiment procedures were in accordance with the guidelines of ASP LIG-16 approved by the Institutional Animal Care and Use Committee (IACUC) of NIAID and #01022 approved by The Jackson Laboratory Institutional Animal Care and Use Committee. BXSB.*Yaa* males and control animals were monitored daily for the development of enlarged lymph nodes. The animals were also monitored for splenomegaly by occasional palpitations. Once BXSB.*Yaa* mice were diagnosed with visible signs of disease, they were sacrificed immediately by CO_2_ asphyxiation along with age matched BXSB female controls.

### Cell isolation and culture

Single cell suspensions were prepared from spleen, bone marrow (BM), lymph nodes and peritoneal washouts. Specific cell populations were isolated using standard protocols. CD43^-^ B cells were isolated by CD43 (Ly-48) microbeads (Miltenyi) using the negative selection method. Splenic dendritic cells (DCs) were isolated using a mouse dendritic cell enrichment set (BD Biosciences) using the manufacturer’s protocol. BM-derived dendritic cells (BM-DCs) were generated by culturing BM cells (10^6^/ml) with GM-CSF (40ng/ml) (Peprotech) and IL4 (20ng/ml) (Peprotech). Media were replenished with fresh cytokine-containing media on days 3, 7 and 9 of culture. Cells were harvested on day 11 and purity was determined by flow cytometry to be 94–95%. Peritoneal macrophages were obtained by adhering peritoneal washout cells to tissue culture plates for 4–5 hours. The non-adherent fraction was removed by repeated washing with sterile ice cold PBS and adherent cells were used for experiments. CD115^+^ monocytes were isolated by positive selection of CD115^+^ cells using a CD115 microbead kit (Miltenyi) following the manufacturer’s protocol. FcR blocking was performed using the 2.4G2 monoclonal antibody (BD Biosciences) as necessary.

Specific cell populations were cultured in the presence of the TLR7 agonist, Imiquimod (R837; Invivogen), for 24–48 hours in complete RPMI-1640 medium (Quality Biological) (RPMI-1640 medium supplemented with 10% FBS (Quality Biological, Lot#205C13), sodium pyruvate, non-essential amino acids, penicillin, streptomycin (all from Quality Biological) and 2-mercaptoethanol (GIBCO)). Cells or culture supernatants were harvested for further studies. In some experiments, B cells were cultured in the presence of anti-BCR antibody (2μg/ml; F(ab’)2 fragment goat anti-mouse IgM, μ chain specific; Jackson Immunoresearch), IFNα or IFNβ (20ng/ml; PBL Biomedical Laboratories).

### Flow cytometry (FACS)

Standard multiparameter FACS analyses were performed using a BD LSRII instrument and antibodies listed in S1 Table. Data were collected using FACS Diva software and analyzed using FlowJo software (Tree Star). Doublet discrimination was performed and viable cells were gated using the propidium iodide exclusion method. B cell activation was assessed using surface expression of CD40, MHCII, ICOSL and CD86. Total splenic B cells were defined as B220^+^IgM^+^ cells and expression levels of IL21R and FAS were measured in terms of mean fluorescence intensity (MFI). Marginal zone B cells were defined as CD19^+^IgM^+^B220^+^CD5^-^CD23^-^CD21^+^, whereas follicular B cells were defined as CD19^+^IgM^+^B220^+^CD5^-^CD23^hi^CD21^lo/-^. Germinal center (GC) B cells were defined as B220^+^IgM^+^PNA^hi^FAS^+^GL7^+^ cells. T_FH_ cells were defined as CD4^+^ICOS^hi^CXCR5^+^PD1^+^ cells.

### ELISA

(a) IL6 and IL21 ELISA. Culture supernatants (neat) or serum (1:20) were added to 96-well ELISA plates (Costar) coated with either purified anti-mouse-IL6 antibody (BD Biosciences) or anti-mouse IL21 antibody (Peprotech). IL6 or IL21 were captured by secondary biotinylated anti-mouse IL6 antibody (BD Biosciences) or anti-mouse IL21 antibody (Peprotech) followed by streptavidin-HRP/TMB (Sigma/Invitrogen) for colorimetric estimation. Washing with PBS-Tween-20 (0.5%) was followed at each step. Results were expressed as ng/ml of secreted IL6 or IL21 using two-fold serial dilutions of recombinant murine IL6 (BD Biosciences) or IL21 (Peprotech) as standards.

(b) Antibody isotype ELISA. Unlabeled purified anti-mouse IgG2b (BD Biosciences) and IgG2c (eBioscience) antibodies were coated on 96-well ELISA plates (Costar) in carbonate-bicarbonate buffer (pH = 9.6) overnight at 4°C. Plates were blocked with 2% IgG-free BSA (Jackson ImmunoResearch) for 2 hours at RT and serial dilutions of serum were added. Bound immunoglobulin isotypes were detected using biotin-labeled antibodies to IgG2b (BD Biosciences) and IgG2c (eBioscience) and color development was detected by streptavidin-HRP (Sigma) followed by TMB (Invitrogen). Reactions were stopped using 1N HCl and the OD was read at 450nm. At each step, plates were washed with PBS-Tween-20 (0.5%).

(c) Anti-nuclear antibody (ANA) or anti-dsDNA antibody (anti-DNA) ELISA. ANA and anti-dsDNA estimation was done using a mouse anti-nuclear Ab Total Ig kit and mouse anti-dsDNA Ab Total Ig kit (Alpha Diagnostics) following the manufacturer’s instructions. We used serum dilutions (1:100) on plates pre-coated with extractable nuclear antigens (ENA) for ANA quantitation (Alpha Diagnostics) or purified dsDNA (Alpha Diagnostics) for anti-dsDNA quantitation. Bound total Ig (IgG+IgM+IgA) was detected by HRP-labeled anti-mouse IgG+IgM+IgA (H+L) detection antibody (Alpha diagnostics) and colorimetric estimation was done using TMB substrate. Stop solution was added to terminate the reaction and color development was read at 450nm. All components in the assay were part of the specific kit used.

### Cell proliferation assay

Negatively selected purified B cells were cultured in appropriate conditions in 96-well U-bottom plates (Costar) for 24h. ^3^H-thymidine (0.5 μCi/ well; Perkin Elmer) was added to the cultures and incubated for a further 16h. The cells were then harvested onto glass fiber filter mats (Perkin Elmer) using a Tomtec Harvester. Proliferation was measured as ^3^H- thymidine incorporation using a Perkin Elmer β-scintillation counter. Results were expressed as counts per minute (CPM).

### Statistical analysis

All values in the figures are expressed as mean ± SEM of n observations (where n is indicated in the figure legends). Relevant data sets were compared by one or two way ANOVA, as deemed appropriate. All data were analyzed using the statistical tools of GraphPad Prism Software.

## Results

### BXSB.*Yaa* mice have increased serum levels of IL6 that correlate with humoral disease manifestations

**Fig 1:**
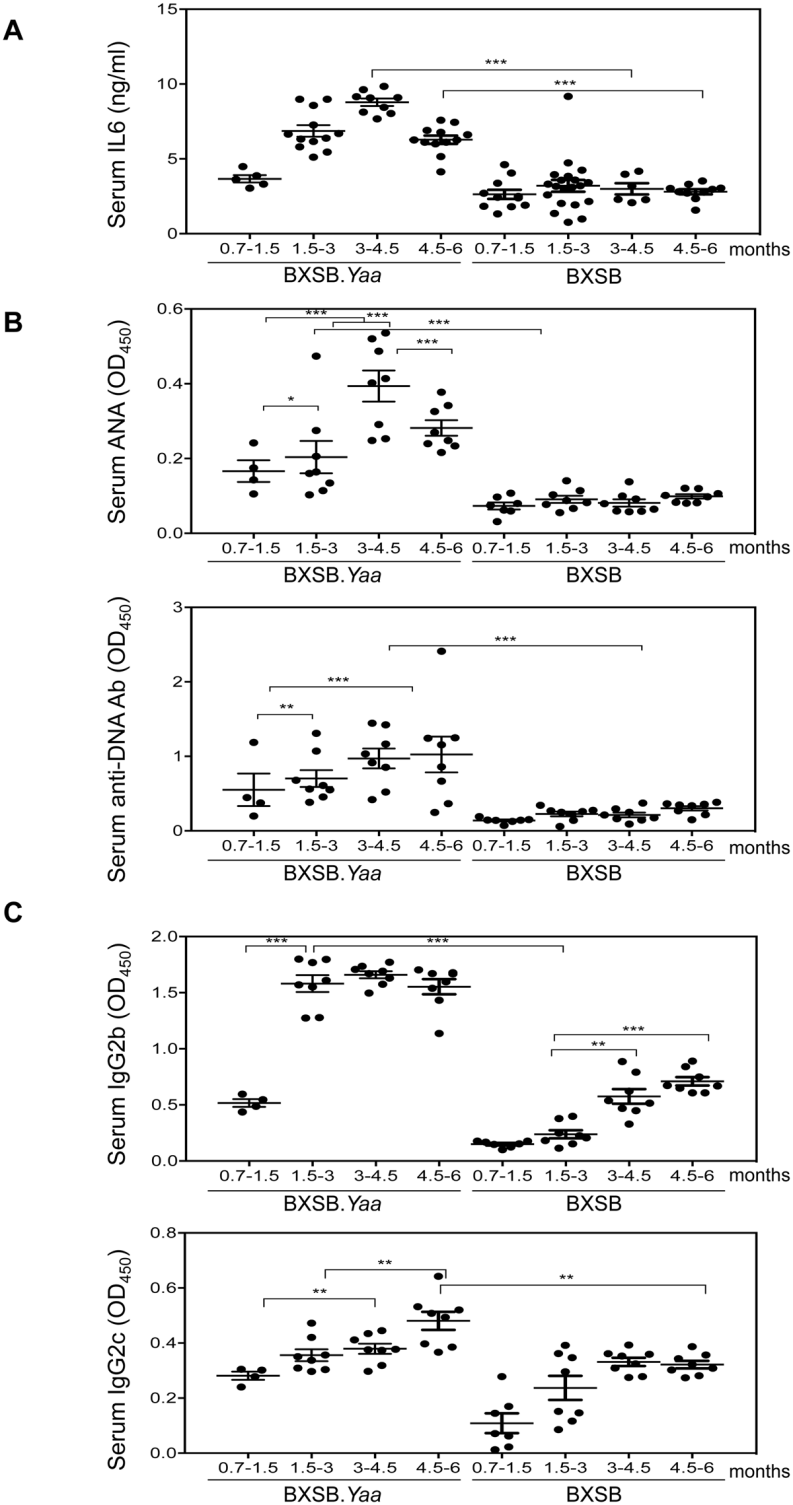
IL6 levels correlate with disease manifestations in BXSB.*Yaa* mice. Sera from BXSB.*Yaa* and BXSB mice of different ages were collected and tested for the levels of (A) IL6 (B) anti-nuclear antibodies (ANA) and anti-dsDNA antibodies, and (C) immunoglobulin isotypes (IgG2b and IgG2c); n = 4–20 mice per age group. Each dot represents one mouse. Horizontal bars represent mean ±SEM. p values are calculated by one-way ANOVA with Tukey’s multiple comparison tests. * p<0.05, ** p<0.01, ***p<0.0001.

To study possible contributions of IL6 to the BXSB.*Yaa* disease, we first compared levels of IL6 in sera from male BXSB.*Yaa* to levels for female BXSB mice, which develop a mild form of autoimmunity when older than 1 year [4, 5]. This comparison eliminated contributions of non-sex chromosome-linked lupus susceptibility genes while emphasizing potential effects of the *Yaa*-determined *Tlr7* duplication. The results demonstrated that IL6 levels were significantly higher in sera of male *Yaa* compared to female mice, even at 1 month of age (Fig 1A). Levels peaked in sera from 3–4.5 month old male mice, the age at which disease symptoms start appearing, but then dropped in older mice. These age-related changes in serum IL6 levels correlated well with changes in serum levels of ANA and anti-dsDNA antibodies that were found at higher levels in male than female BXSB mice, again peaking at 3–4.5 months of age (Fig 1B).

The fact that IL6 is known to augment plasma cell differentiation and immunoglobulin secretion led us to determine if age-related changes in IL6 expression would correlate with changes in serum immunoglobulin levels. Analyses of serum IgG2b showed that levels in male mice increased dramatically between 0.7–1.5 and 1.5–3 months of age and remained equally high thereafter (Fig 1C). Serum IgG2b levels of female mice also increased with age but were always substantially lower than for male BXSB.*Yaa* mice at each time point examined. These same patterns and relationships between sexes were also seen in analyses of serum IgG2c levels (Fig 1C). From these findings, we concluded that IL6 secretion correlates with the humoral progression of disease in BXSB.*Yaa* mice.

### B cells are prominent producers of IL6

**Fig 2:**
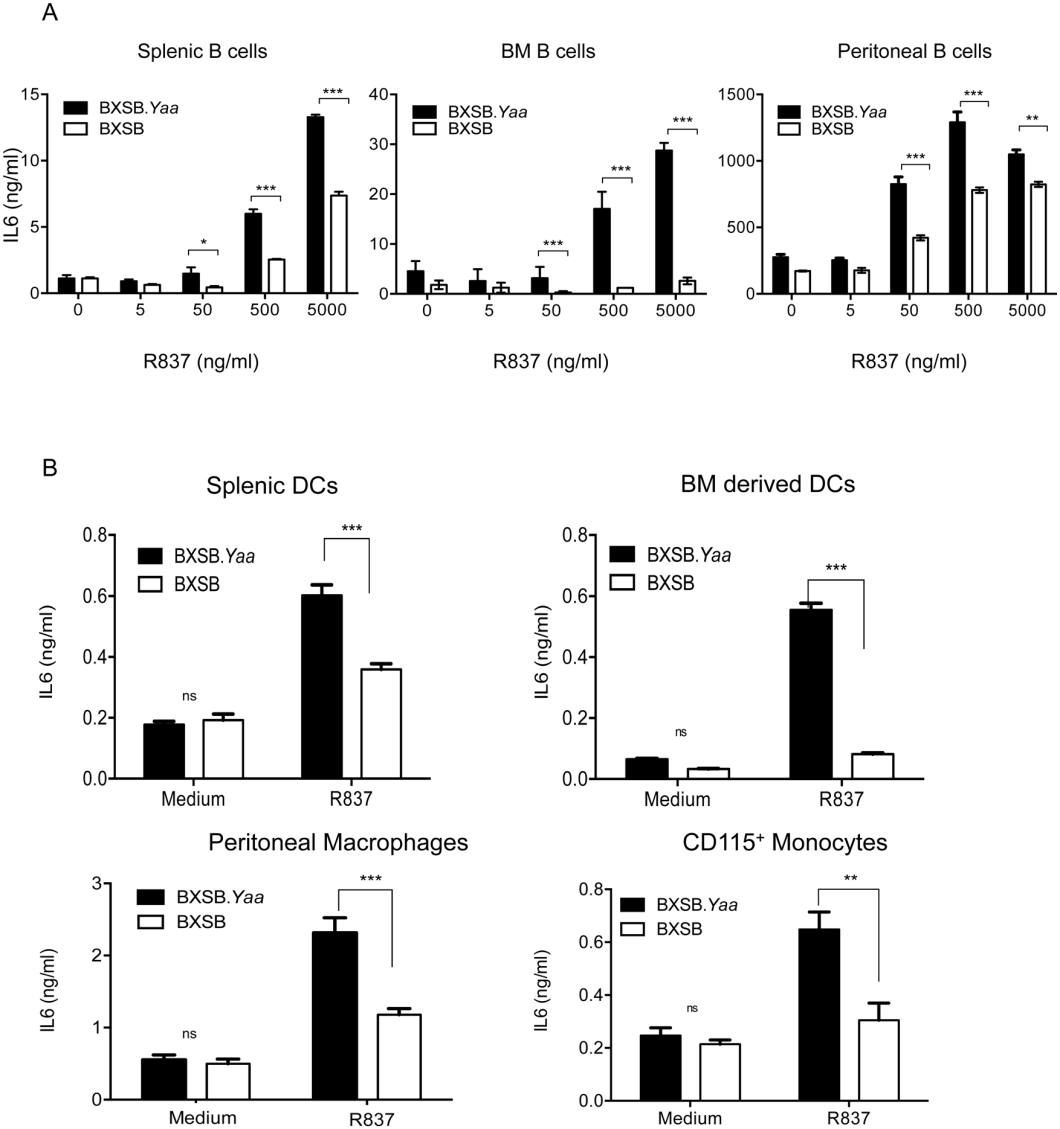
B cells from BXSB.*Yaa* mice are potent producers of IL6. (A) CD43^-^ B cells were purified from spleen, BM and peritoneal lavage of 4–5 month old BXSB.*Yaa* and BXSB mice and stimulated with graded concentrations of Imiquimod (R837). (B) Dendritic cells enriched from spleen (splenic DCs) or differentiated from BM precursors (GMCSF (40ng/ml) and IL4 (20ng/ml) for 9 d with media changed every third day), peritoneal macrophages and CD115^+^ monocytes from spleens of BXSB.*Yaa* and BXSB mice were stimulated with R837 (50ng/ml) *in vitro*. Supernatants were collected after 24 hours and IL6 levels were quantified using a standard sandwich ELISA. Data expressed as mean ± SEM of triplicate wells are representative of 4–6 independent experiments. p values are determined by two-way ANOVA. * p<0.05, ** p<0.01, ***p<0.0001.

Previous studies in mice showed that B cells secrete IL6 in response to stimulation with TLR7 ligands [6, 25, 26]. In addition, it was shown that lymphoblastoid B cells isolated from lupus patients produced increased levels of IL6 [27]. To determine whether B cells from different hematopoietic compartments varied in their ability to produce IL6, we compared levels of IL6 secretion in culture supernatants of CD43^-^ B cells purified from spleen, BM and peritoneal lavage from male and female BXSB mice. The cells were cultured for 24 hours with or without the TLR7 agonist, Imiquimod (R837). We found that B cells from BXSB.*Yaa* mice, regardless of source, secreted significantly higher levels of IL6 than cells from BXSB females when stimulated with R837 (Fig 2A). In addition, the levels of IL6 secreted by unstimulated or R837-stimulated peritoneal B cells were strikingly higher than those secreted by comparable populations of splenic or BM B cells (note differences in scale). It is known that IL6 is also produced by other APCs such as conventional splenic dendritic cells (cDCs), bone marrow derived dendritic cells (BM DCs), peritoneal resident macrophages and CD115^+^ monocytes. We next compared the levels of IL6 secreted by these cell subsets isolated from BXSB.*Yaa* and BXSB mice when stimulated with R837 *in vitro*. We found that IL6 levels secreted by each stimulated population were significantly higher in BXSB.*Yaa* compared with female BXSB control mice; however, these levels were markedly lower than those produced by any of the B cell populations studied (Fig 2B). This led us to conclude that IL6 secreted primarily by B cells in response to enhanced TLR7 expression as the result of *Yaa* is likely to be an important contributor to development of SLE-like disease in BXSB.*Yaa* mice.

### BCR and TLR7 signaling pathways cooperate to induce IL6 secretion by B cells

**Fig 3:**
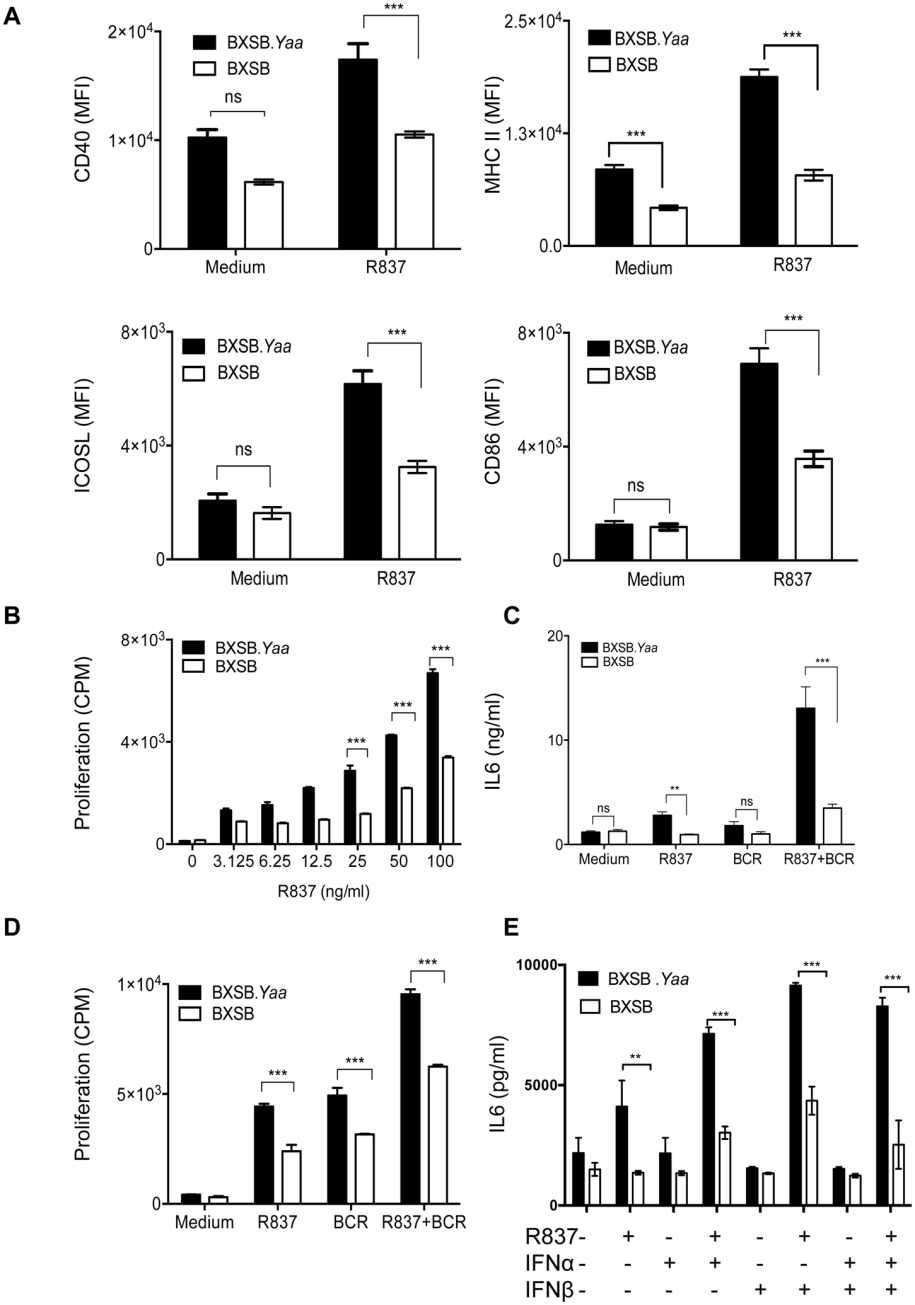
BXSB.*Yaa* B cells exhibit an activated phenotype. (A) Purified CD43^-^ B cells from 4–5 month old BXSB.*Yaa* and BXSB mice were stimulated with R837 (50ng/ml) for 24 hours and stained for surface expression of the activation markers—CD40, MHCII, ICOSL and CD86. Data are expressed as mean ± SEM of mean fluorescence intensity (MFI) and are pooled from 4–5 independent experiments. (B) Purified CD43^-^ B cells from 4–5 month old BXSB.*Yaa* and BXSB mice were stimulated with graded doses of R837 for 24 hours. ^3^H thymidine (0.5μCi/well) was added and its incorporation was determined after 16 hours by liquid scintillation counting. Proliferation was determined as counts per minute (CPM). (C-D) CD43^-^ B cells were stimulated with R837 (50ng/ml) in the presence and absence of anti-BCR antibody. IL6 secretion (C) and B cell proliferation (D) were determined as described earlier. (E) Purified B cells from 4–5 month old BXSB.*Yaa* and BXSB mice were cultured in the presence or absence of R837 (50ng/ml); IFNα and IFNβ (40U/ml), either alone or in combination, for 24h. Supernatants were collected and quantified for IL6 levels by standard sandwich ELISA. All data (B-E) are expressed as mean ± SEM of triplicate wells and are representative of 2–3 independent experiments. P values are determined by two-way ANOVA. * p<0.05, ** p<0.01, ***p<0.0001.

The understanding that B cells are the primary producers of IL6 in BXSB.*Yaa* mice prompted us to determine whether their B cells are spontaneously activated. We found that the basal expression levels of activation markers for purified splenic B cells cultured in medium alone tended to be higher for cells from *Yaa* males compared with those from female BXSB mice, but reaching significance only for expression of MHCII (Fig 3A). These differences were greatly magnified after stimulation with R837 (Fig 3A). From this, we concluded that B cells from BXSB.*Yaa* mice are activated in the absence of overt stimulation and were substantially more responsive to activation by ligation of TLR7.

Spontaneous activation of BXSB.*Yaa* cells may be attributed to the additional copy of *Tlr7* that leads to B cell-mediated disease progression. Since TLR7 also promotes the proliferation, migration and terminal differentiation of murine B cells [28, 29], we further compared the proliferative responses of purified splenic B cells from male *Yaa* and female BXSB mice to R837 stimulation *in vitro*. B cells from both BXSB.*Yaa* and BXSB mice proliferated in a dose-dependent manner in response to TLR7 ligation, but the proliferative responses of B cells from males were always higher than those from cells from female mice (Fig 3B).

Previous studies have shown that co-stimulation through the BCR and TLR synergizes in activating B cells and that these activated B cells secrete elevated levels of IL6 [26, 29, 30]. To determine if B cell activation in BXSB.*Yaa* mice is a consequence of cooperative BCR and TLR signaling, we stimulated purified splenic B cells from male and female BXSB mice with R837 or the F(ab’)2 fragment of anti-IgM antibody (BCR), either alone or in combination. Supernatants of the cultures were assayed for IL6 levels (Fig 3C) and the cells were also assessed for proliferation (Fig 3D). We found that combined stimulation through TLR7 and the BCR synergistically augmented IL6 secretion by male and female B cells (Fig 3C). In addition, co-stimulation also enhanced the proliferative responses of both male and female B cells to these stimuli with the responses of male cells being significantly greater (Fig 3D). Collectively, these results indicated that B cells from BXSB.*Yaa* mice have a spontaneously activated phenotype, which is enhanced by co-engagement of the BCR and TLR7, a combination that also induced enhanced IL6 production and proliferation.

### IFN1 enhances B cell activation driven by co-ligation of TLR7 and the BCR

Previous studies showed that IFN1 sensitizes B cells to activation by a number of stimuli [31–33]. In addition, an “IFN signature”, marked by heightened expression of a series of IFN-stimulated genes, is associated with flares in human SLE [34–36]. We recently found that purified B cells from T cell-deficient BXSB.*Yaa* mice as young as 4 weeks of age have a lupus-like “IFN signature” that develops in the absence of IL21. This signature is induced by expression of IFN1 by plasmacytoid DC (pDC) via a signaling pathway that is at least partially dependent on TLR7 (*manuscript in preparation*). To determine if IFN1 would affect the responsiveness of B cells from BXSB.*Yaa* mice, we cultured purified splenic B cells from both male and female BXSB mice in the presence or absence of R837, IFNα and IFNβ. We found that stimulation of B cells with IFN1 in the presence of a TLR7 ligand significantly enhanced B cell production of IL6 with male cells being significantly more responsive than female BXSB cells (Fig 3E). Moreover, additional stimulation through the BCR further stimulated IL6 secretion, indicating that signaling through the IFNAR, TLR7 and the BCR have a cooperative effect on IL6 production in BXSB.*Yaa* mice (S1 Fig). From this, we conclude that stimulation of B cells by both innate (IFN1, TLR7) and adaptive (BCR) signals collectively enhance IL6 secretion in BXSB.*Yaa* mice.

### Abrogating IL6 signaling delays disease in BXSB.*Yaa* mice

**Fig 4:**
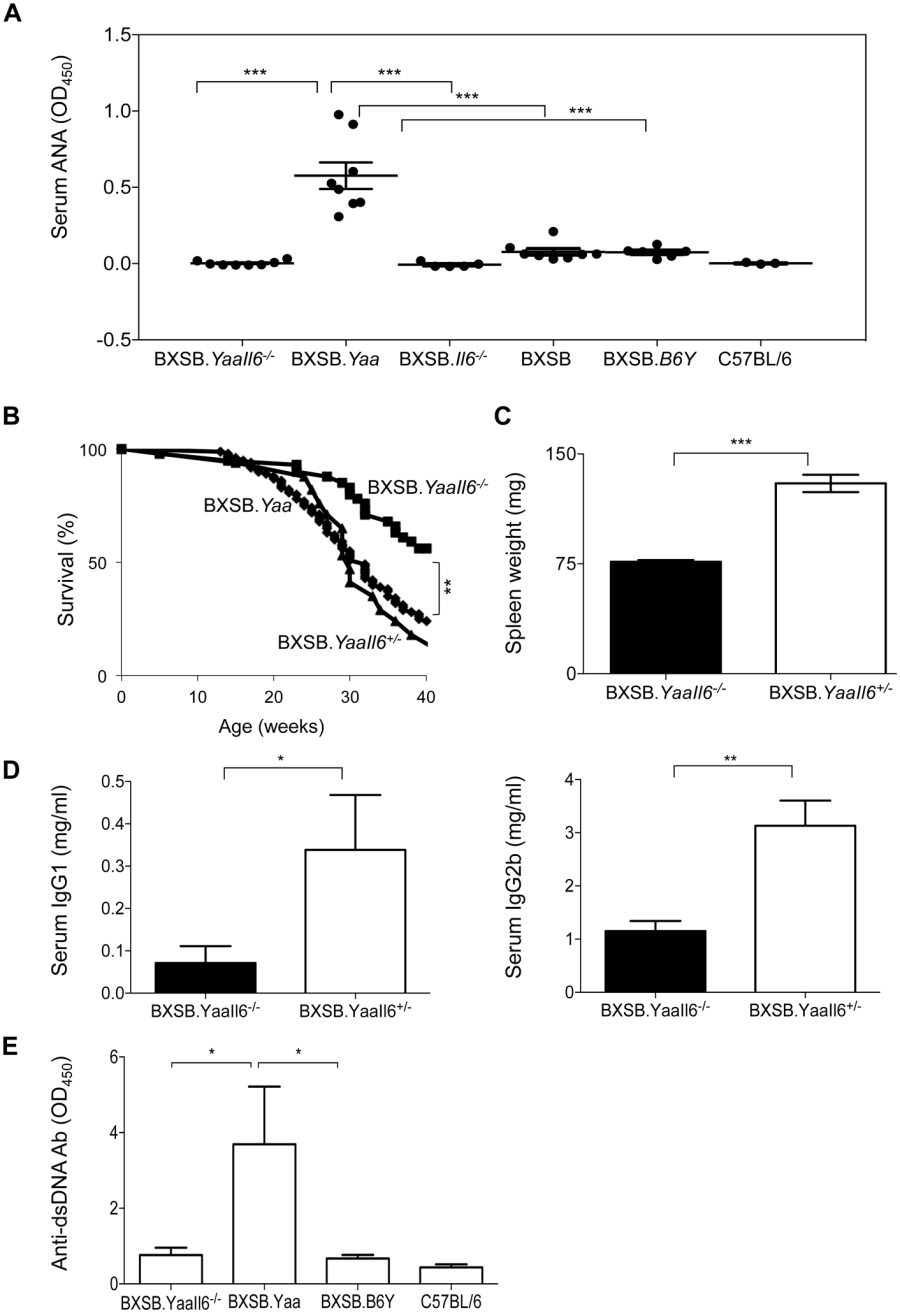
IL6-deficient BXSB.*Yaa* mice have reduced disease manifestations. (A) Sera from 6-month old BXSB.*Yaa*.*Il6*^*-/-*^, BXSB.*Yaa*, BXSB.*Il6*^*-/-*^, BXSB, BXSB.*B6Y* and C57BL/6 mice were quantified for the presence of ANA by ELISA (n = 3–10 mice per group). Each dot represents one mouse. Horizontal bars indicate mean ± SEM and p values are calculated by one-way ANOVA with Tukey’s multiple comparison tests. (B) Survival curve analysis of BXSB.*Yaa*.*Il6*^*+/-*^ (n = 17), BXSB.*Yaa*.*Il6*^*-/—*^(n = 41) and archival data of BXSB.*Yaa* (n = 93) mice. (C-E) IL6-competent and—deficient BXSB.*Yaa* mice (n = 8–10) were compared for disease manifestations including spleen weight (C), serum IgG1 and IgG2b (D) and serum anti-dsDNA antibody titers (E). Bars represent mean ± SEM of observations from individual mice and p values are calculated using two-way ANOVA. * p<0.05, ** p<0.01, ***p<0.0001.

To directly assess the importance of IL6 to the SLE-like disease of BXSB.*Yaa* mice, we generated BXSB.*Yaa* mice homozygous for a null allele of *Il6* (BXSB.*Yaa*.*Il6*^-/-^) and studied them for disease development. First, we compared autoantibody levels in sera from 6–10 month old BXSB.*Yaa*.*Il6*^-/-^, BXSB.*Yaa*, and BXSB.*B6Y* consomic controls carrying a B6 Y chromosome as well as BXSB.*Il6*^-/-^ females. We found that ANA levels in sera of IL6-deficient BXSB.*Yaa* mice were significantly lower than those of BXSB.*Yaa* mice and that they were comparable to those of female BXSB mice and BXSB.*B6Y* consomic controls. (Fig 4A). This observation suggested that IL6 signaling is a major contributor to the development of autoantibodies. To further address this issue, we compared the survival and disease characteristics of BXSB.*Yaa*.*Il6*^-/-^, BXSB.*Yaa*.*Il6*^+/-^ and BXSB.*Yaa* mice. We found that the survival of BXSB.*Yaa*.*Il6*^-/-^ mice was significantly prolonged compared to that of BXSB.*Yaa* and BXSB.*Yaa*.*Il6*^+/-^ mice (Fig 4B). Further studies demonstrated that spleen weights (Fig 4C) and serum levels of IgG2b and IgG1 (Fig 4D) were significantly lower in BXSB.*Yaa*.*Il6*^-/-^ than BXSB.*Yaa*.*Il6*^+/-^ mice. In addition, serum levels of anti-dsDNA antibodies were significantly lower in sera of BXSB.*Yaa*.*Il6*^-/-^ than BXSB.*Yaa* mice and were comparable to levels in sera of BXSB.*B6Y* consomic controls and normal B6 mice (Fig 4E).

**Fig 5:**
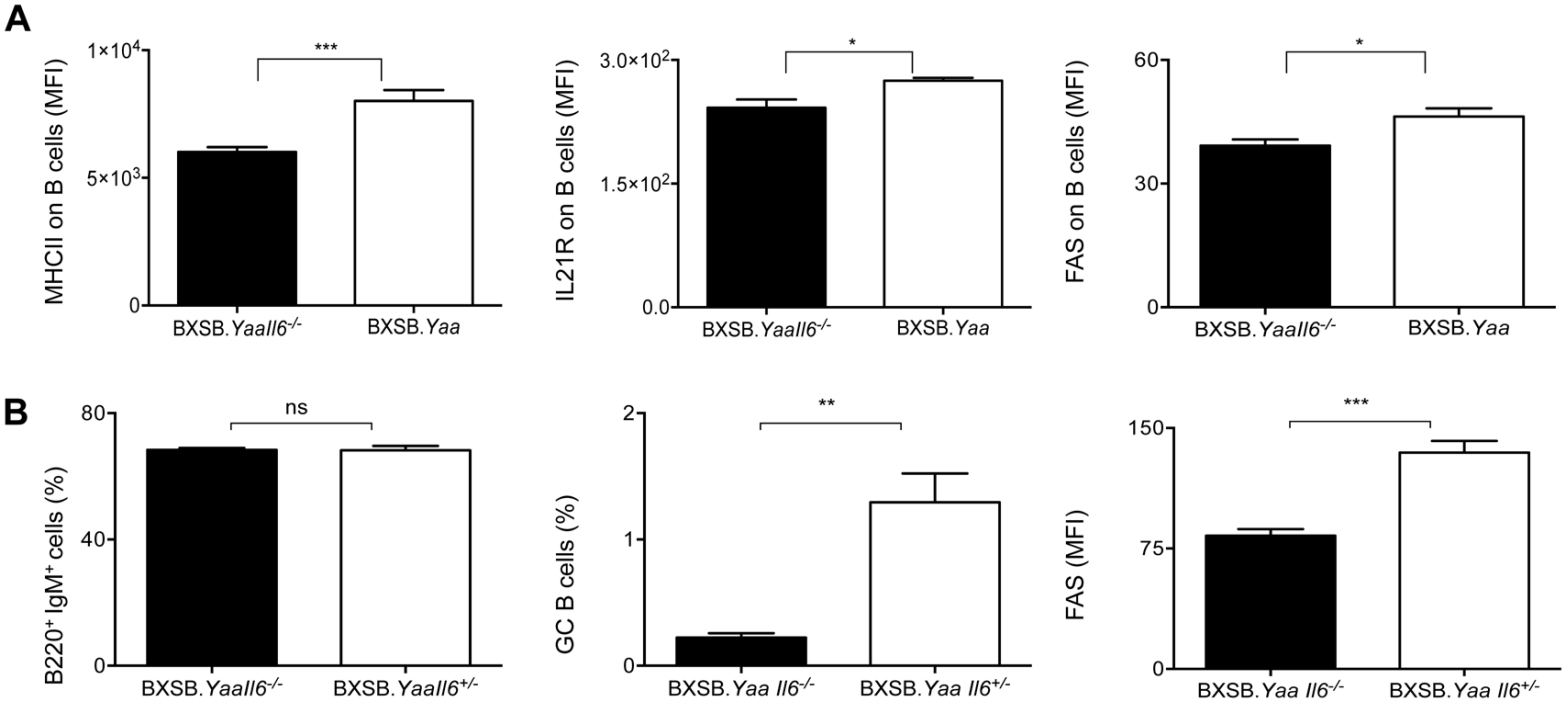
Early activation of B cells in BXSB.*Yaa* mice is reduced in the absence of IL6. 6–7.5 weeks old BXSB.*Yaa* (n = 5) and BXSB.*Yaa*.*Il6*^*-/-*^ (n = 8) mice were bled and (A) cells were stained with fluorescent-labeled antibodies to B220, IgM, MHCII, IL21R and FAS and analyzed by FACS. Expression of MHCII, IL21R and FAS on viable single cells gated on B220^+^IgM^+^ is determined as MFI. (B) The frequencies of splenic total B cells (B220^+^IgM^+^) and GC B cells (B220^+^IgM^+^PNA^hi^FAS^+^GL7^+^) were determined and expressed as percentages of total cells. Expression levels of FAS on GC B cells are depicted as mean fluorescence intensity (MFI). Data are expressed as mean ± SEM of individual values obtained from each mouse and statistical analysis is done using two tailed unpaired t test. * p<0.05, ** p<0.01, ***p<0.0001.

To determine if the cellular effects of IL6 deficiency can be detected in young mice, we first characterized cells in the blood of 6 week old BXSB.*Yaa*.*Il6*^-/-^ and BXSB.*Yaa*.*Il6*^+/-^ mice. FACS analyses of different activation markers revealed that levels of expression of MHCII, IL21R and FAS were lower on B cells of BXSB.*Yaa*.*Il6*^-/-^ than BXSB.*Yaa* mice (Fig 5A). Interestingly, in studies of spleens, the frequencies of total B220^+^IgM^+^ B cells were equivalent for BXSB.*Yaa*.*Il6*^-/-^, BXSB.*Yaa* and BXSB.*B6Y* mice (Fig 5B, left panel and data not shown). In contrast, the frequencies of splenic GC B cells and levels of FAS expression on B cells were significantly lower for BXSB.*Yaa*.*Il6*^-/-^ than BXSB.*Yaa*.*Il6*^+/-^ mice (Fig 5B and S2 Fig). These data clearly indicated that the effects of IL6 deficiency could be detected early in the life of BXSB.*Yaa* mice, consistent with an important role in disease initiation.

Earlier studies showed that BXSB.*Yaa* mice have reduced frequencies of marginal zone B cells [37] caused, at least in part, by high levels of IL21 expression [9]. We observed that in the absence of IL6 signaling, the frequencies of marginal zone B cells were increased in BXSB.*Yaa Il6*^-/-^ compared with BXSB.*Yaa* mice, though not significantly. However, the increased frequencies of marginal zone B cells were not comparable to the frequencies seen with consomic BXSB.*B6Y* controls suggesting that enhanced expression of IL6 in addition to IL21 contributes to the reduction in the marginal zone B cell compartment of BXSB.*Yaa* mice (S3A and S3B Fig). Since activated monocytes and granulocytes are some of the other known disease manifestations of BXSB.*Yaa* mice [38–40], we asked whether ablation of IL6 signaling had a significant impact on these cell populations. We found that both CD11b^+^ and Gr-1^+^ cells had significantly lower expression of MHCII in BXSB.*Yaa*.*Il6*^-/-^ mice as compared to BXSB.*Yaa* mice (S3C Fig) suggesting that ablation of IL6 signaling reduced levels of activated monocytes and granulocytes in BXSB.*Yaa* mice.

Taken together, these data support our suggestion that interruption of IL6 signaling has a significant impact on disease development from early in life that ultimately results in increased survival of BXSB.*Yaa* mice.

### IL6 acts upstream of IL21 in disease progression caused by *Yaa*

**Fig 6:**
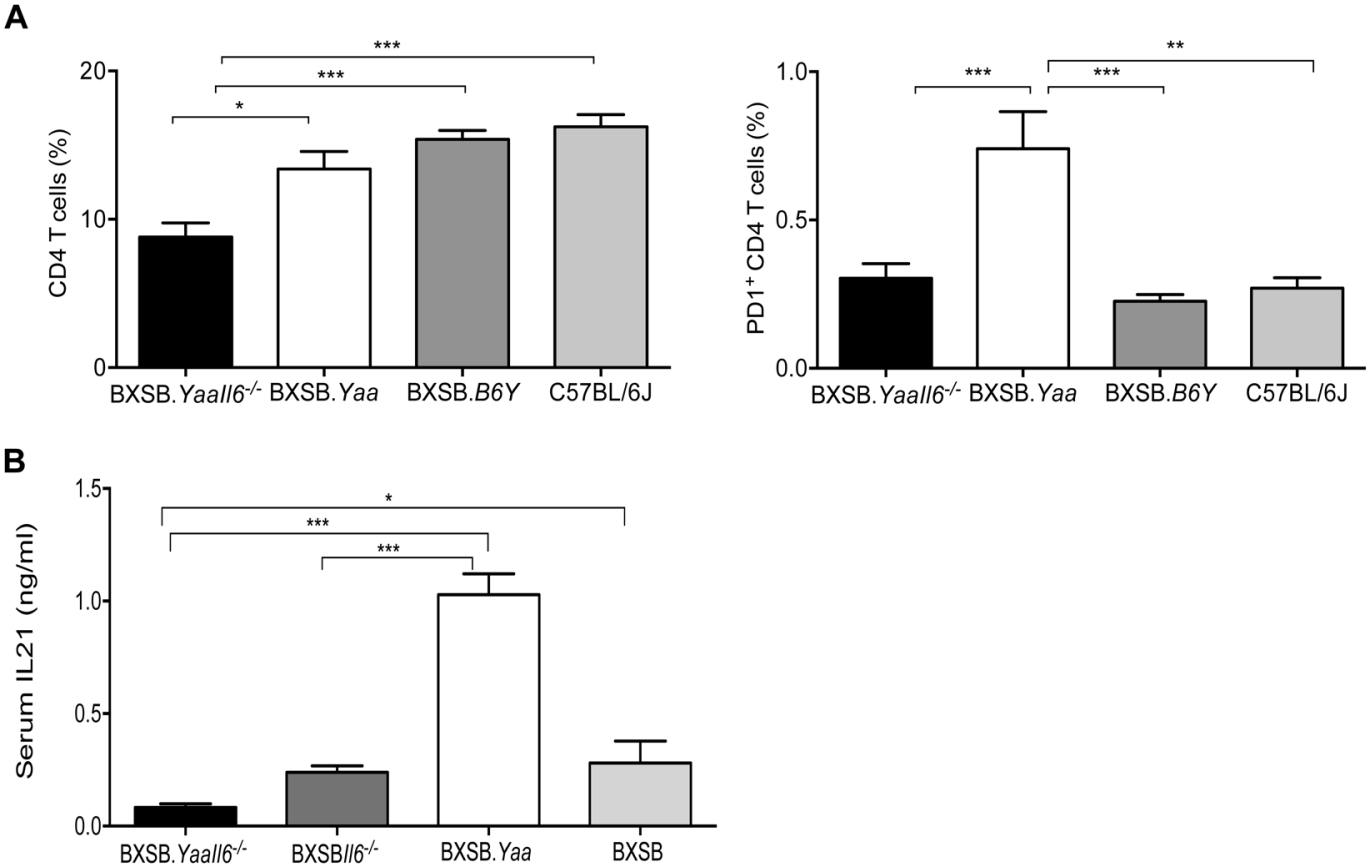
IL6 deficiency reduces the T_FH_ population in BXSB.*Yaa* mice. (A) Splenocytes from BXSB.*Yaa*.*Il6*^*-/-*^ (n = 8) BXSB.*Yaa* (n = 5), BXSB.B6Y (n = 6) and C57BL/6J (n = 3) mice aged 6–9 weeks were stained with labeled antibodies to CD4, ICOS and PD1. Percentages of CD4^+^ T cells (CD4^+^) and T_FH_ cells (CD4^+^ICOS^hi^PD1^+^) cells were determined by FACS and expressed as mean ± SEM. (B) Sera from BXSB.*Yaa*.*Il6*^*-/-*^ (n = 12; 1.5–5 months old), BXSB.*Il6*^*-/-*^ (n = 5; 3–5 months old); BXSB.*Yaa* (n = 5; 3–5 months old) and BXSB (n = 5; 3–5 months old) mice were quantified for IL21 by sandwich ELISA. Data is expressed as mean ± SEM from observations from each mouse. Statistical analysis was done using one-way ANOVA with Tukey’s multiple comparison test. * p<0.05, ** p<0.01, ***p<0.0001.

We previously reported that IL21, the signature cytokine secreted by CD4^+^ T follicular helper (T_FH_) cells, is a critical determinant of the disease of BXSB.*Yaa* mice [9–11]. Previous studies reported that IL6 promotes the secretion of IL21 and that optimal T_FH_ differentiation is mediated by both IL6 and IL21 [15, 41]. In addition, our observation that BXSB.*Yaa* mice have expanded populations of T_FH_ prompted us to examine whether ablation of IL6 would affect the expansions of T_FH_ cells. We found, first, that the frequencies of total CD4^+^ T cells were significantly reduced in spleens of IL6-deficient BXSB.*Yaa* mice as compared to normal BXSB.*Yaa* mice (Fig 6A, left panel, S4A Fig). Second, IL6 deficiency impacted the frequencies of T_FH_ by 6 weeks of age as evidenced by the significant reduction in the frequencies of PD1^+^CD4^+^ T cells in spleens of IL6-deficient BXSB.*Yaa* mice (Fig 6A, right panel, S4B Fig) as compared to BXSB.*Yaa* mice or consomic BXSB.*B6Y* controls. To ascertain whether the reduction in CD4^+^ T_FH_ cell numbers required the autosomal SLE predisposition background of BXSB, we determined the effects of an IL6 deficiency in B6.*Yaa* mice, which develop a late onset autoimmune disease due to *Yaa*. We found that B6.*Yaa Il6*^*-/-*^ mice analyzed at 40 weeks of age had reduced frequencies of CD4^+^ T_FH_ cells (S5A and S5B Fig) as evidenced by the reduced expression of ICOS and PD1 on CD4^+^ T cells of B6.*YaaIl6*^*-/-*^ mice when compared with age matched WT controls (S5C and S5D Fig). Finally, we quantified serum IL21 levels in IL6-deficient BXSB.*Yaa* mice from 6 weeks to 5 months of age and found that the levels were significant decreased at both time points (Fig 6B). This indicates that IL6 acts upstream of IL21 in the early phase of BXSB.*Yaa* disease by limiting the development of T_FH_ which are the primary source of IL21. From this we concluded that IL6 acts to promote and maintain the development of T_FH_ that are the primary source of IL21 required for the SLE-like manifestations caused by the *Yaa* mutation.

## Discussion

IL6 is a pleiotropic proinflammatory cytokine that normally induces terminal differentiation of B cells into plasma cells and memory B cells. IL6 also affects T cells by transcriptionally activating naïve CD4^+^ T cells to become T_FH_ [42]. However, heightened expression of IL6 has also been associated with a range of pathologies including a variety of autoimmune diseases in humans and mice. A close association between expression of IL6 and progression of lupus-like diseases has been described for several mouse models of SLE. Studies of autoimmune MRL.*Fas*^*lpr*^ mice showed that serum levels of IL6 and the IL6 receptor (IL6R) increased in an age-dependent manner [18–20]. Treatment with an IL6 neutralizing antibody led to reduced production of autoantibodies in old (NZB x NZW)F1 mice [43]. In the pristane-induced model of SLE, production of autoantibodies was abrogated in mice deficient in IL6 [44]. Conversely, treatment of (NZB x NZW)F1 mice with IL6 accelerated disease progression [45]. Finally, several studies of human SLE have suggested a prominent role for IL6 in B cell hyperactivity and immunopathology in that elevated serum levels of IL6 were associated with disease activity or levels of autoantibodies [21, 22]. It was also reported that B cells from patients with SLE spontaneously produced high levels of IL6 and expressed the IL6R [46, 47].

It is known that IL6 production by both human and murine B cells is enhanced following cooperative signaling through TLR7 and CD40 [26]. Moreover, autoreactive B cells can be activated by simultaneous ligation of TLR7 and the BCR [48]. TLR7 recognizes ssRNA and signals through MyD88 resulting in the activation of B cells in an NF-κB-dependent manner [49]. TLR7 is also considered to be an important contributor to the development of human lupus [50–53]. The recombinant inbred mouse, BXSB.*Yaa*, develops an accelerated SLE-like disease due to the translocation of a telomeric region of the X chromosome to the Y chromosome, resulting in the duplication of at least 16 genes including *Tlr7* in male mice [2, 3]. The duplication of *Tlr7* has been shown to be primarily determinant responsible for the disease of BXSB.*Yaa* mice. *Tlr7* is widely considered to be the genetic driver of overt SLE-like disease in the presence of other lupus susceptibility alleles in mice and genome wide association studies identified TLR7 as a risk factor for SLE in humans [50–53].

The present study was designed to address the extent to which IL6 contributes to the autoimmune disease caused by *Yaa* in BXSB mice and its cellular sources. We clearly established that BXSB.*Yaa* mice have elevated serum levels of IL6 in association with increasing disease manifestations of hypergammaglobulinemia and levels of ANA in an age-dependent manner. These findings are strongly supported by our results showing that IL6-deficient BXSB.*Yaa* mice had greatly reduced levels of class-switched immunoglobulins and autoantibodies and lived significantly longer. Further, *Yaa* causes B cells in these mice to become activated, highly responsive to TLR7, BCR and TLR7 + BCR stimulation, and to secrete high levels of IL6, all of which is consistent with B cells being the primary source of IL6 that promotes BXSB.*Yaa* disease. In addition, B cells also express type I interferon receptors (IFNAR). IFN1 has been shown to sensitize B cells to be more responsive to BCR signals, enhance their proliferation and survival and to augment antibody responses while promoting isotype switching [54–56]. Our results indicate that BXSB.*Yaa* B cells secrete large amounts of IL6 when stimulated simultaneously through IFNAR, TLR7 and the BCR suggesting that IL6 is critical to development of disease at a very early stage.

Our previous studies established that IL21-IL21R signaling to B cells resulting in expanded populations of CD4^+^ T_FH_ is a critical factor in driving disease in these mice, while IL21-IL21R signaling to CD8^+^ T and NK cells restricts disease progression [9–11]. Thus, the development of T_FH_ that secrete high levels of IL21 is a central component of the BXSB.*Yaa* disease. Here we found that the frequency of CD4^+^ T_FH_ cells and the serum levels of IL21 were significantly decreased in IL6-deficient BXSB.*Yaa* mice. Moreover, the involvement of IL6 in supporting the expansion of T_FH_ was evident in BXSB.*Yaa* mice at early age mice. IL6 has been shown to be an important factor in the activation of BCL6, which is the critical transcription factor in the differentiation of naïve CD4^+^ T cells to T_FH_ [14, 15]. IL6 has been shown to support T_FH_ differentiation even at their earliest stages of lineage commitment [42, 57, 58]. The abundance of IL6 secreted mainly by activated B cells in a TLR7-dependent manner suggests that they are the major source of IL6 that drives the expansion of IL21-producing T_FH_ in BXSB.*Yaa* mice.

Taken together, the results of this study define an important role for IL6 in the development and progression of the SLE-like disease of BXSB.*Yaa* mice, suggesting that IL6 signaling could provide an important point for therapeutic intervention. A human open-label phase I trial in SLE patients of Tocilizumab, a monoclonal antibody that inhibits binding of IL6 to its receptor, revealed improvements in disease activity and reduced levels of serum autoantibodies [59]. However, the patients also experienced an increased number of infections suggesting that changes in the protocol or adoption of new approaches to blocking IL6 signaling were in order. In another double blind phase II trial, SLE patients treated intravenously with Sirukumab, a fully human anti-IL6 monoclonal antibody, suffered from some minor respiratory infections [60]. If direct interference with IL6-IL6R signaling is not possible due to adverse effects, several JAK inhibitors such as Tofacitinib and Ruxolitinib that prevent downstream signaling from the IL6R may be worth investigating for therapeutic purposes. Since IL6 production is enhanced by co-stimulation through TLR7 and the BCR, other therapeutic interventions could include inhibitors of TLR7, IRF7 and STAT activity.

## Supporting Information

S1 Fig Concomitant signaling through TLR, BCR and type I interferon enhances IL6 secretion by BXSB.*Yaa* B cells. Purified B cells from BXSB.*Yaa* and BXSB mice were cultured in the presence or absence of R837 (50ng/ml); anti-BCR antibody (2g/ml); IFN and (40U/ml), either alone or in combinations, for 24h. Supernatants were collected and quantified for IL6 levels by standard sandwich ELISA method. Data is expressed as mean ± SEM of triplicate wells and is representative of two independent experiments.
(TIF)

S2 Fig Gating strategy for germinal center B cells. Splenocytes from BXSB.*Yaa*, BXSB.*Yaa* and BXSB mice were isolated and stained with anti-mouse antibodies to identify germinal center B cells (B220^+^GL7^+^Fas^+^).
(TIF)

S3 Fig Abrogating IL6 signaling diminishes marginal zone B cell frequencies and activation of monocytes. Splenocytes from BXSB.*Yaa*.*Il6*^-/-^, BXSB.*Yaa* and consomic BXSB.*B6Y* mice were isolated and stained with anti-mouse antibodies to determine (A-B) marginal zone B cells (CD19^+^IgM^+^B220^+^CD5^-^CD23^-^CD21^+^) and (B) MHCII expression on CD11b^+^ and Gr-1^+^ cells.
(TIF)

S4 Fig Gating strategy for CD4 T_FH_ cells. Splenocytes from B6.*Yaa*.*Il6*^-/-^ and B6.*Yaa Il6*^+/-^ mice were isolated and stained with anti-mouse antibodies to determine (A) CD4T_FH_ cells (PD1^+^ ICOS^hi^ CD4^+^) and (B) PD1^+^ CD4^+^ cells.
(TIF)

S5 Fig IL6 acts upstream of IL21 in the disease progression of BXSB.*Yaa* mice. Splenocytes from B6.*Yaa*.*Il6*^-/-^ and B6.*Yaa Il6*^+/-^ mice were isolated and stained with anti-mouse antibodies. (A) FACS plots determine ICOS vs PD-1 expression on CD4^+^ T cells. Numbers in the plots represent percentage of total cells. Bar diagrams represent (B) percentages of CD4^+^ T cells (C) percentages of CD4T_FH_ (PD1^+^ ICOS+ CD4^+^) cells (D) ICOS and PD-1 expression (MFI) on CD4 T cells. (B) and (C) represent frequencies of parent population. Data is mean ± SEM of 7–8 mice per group. P values were determined by two way ANOVA.
(TIF)

S1 Table List of Antibodies used in FACS and ELISA. (DOCX)

## Abbreviations

ANA: antinuclear antibodies
BM-DCs: bone marrow-derived dendritic cells
cDCs: conventional dendritic cells
CPM: counts per minute
FcR: Fc receptor
GC: germinal center
GWAS: genome wide association study
IFN1: Type I interferons
IL6R: Interleukin 6 receptor
IRF7: Interferon regulatory factor 7
SLE: systemic lupus erythematosus
ssRNA: single stranded RNA
T_FH_: CD4^+^ T follicular helper cells
TLR: Toll like receptors
Ts: CD8^+^ T suppressor cells
