# Supplementary figures and images for: Interleukin 6 Accelerates Mortality by Promoting the Progression of the Systemic Lupus Erythematosus-Like Disease of BXSB.Yaa Mice
Source: PLoS One. 2016 Apr 6;11(4):e0153059. doi: 10.1371/journal.pone.0153059 (PMC4822786; doi:10.1371/journal.pone.0153059)

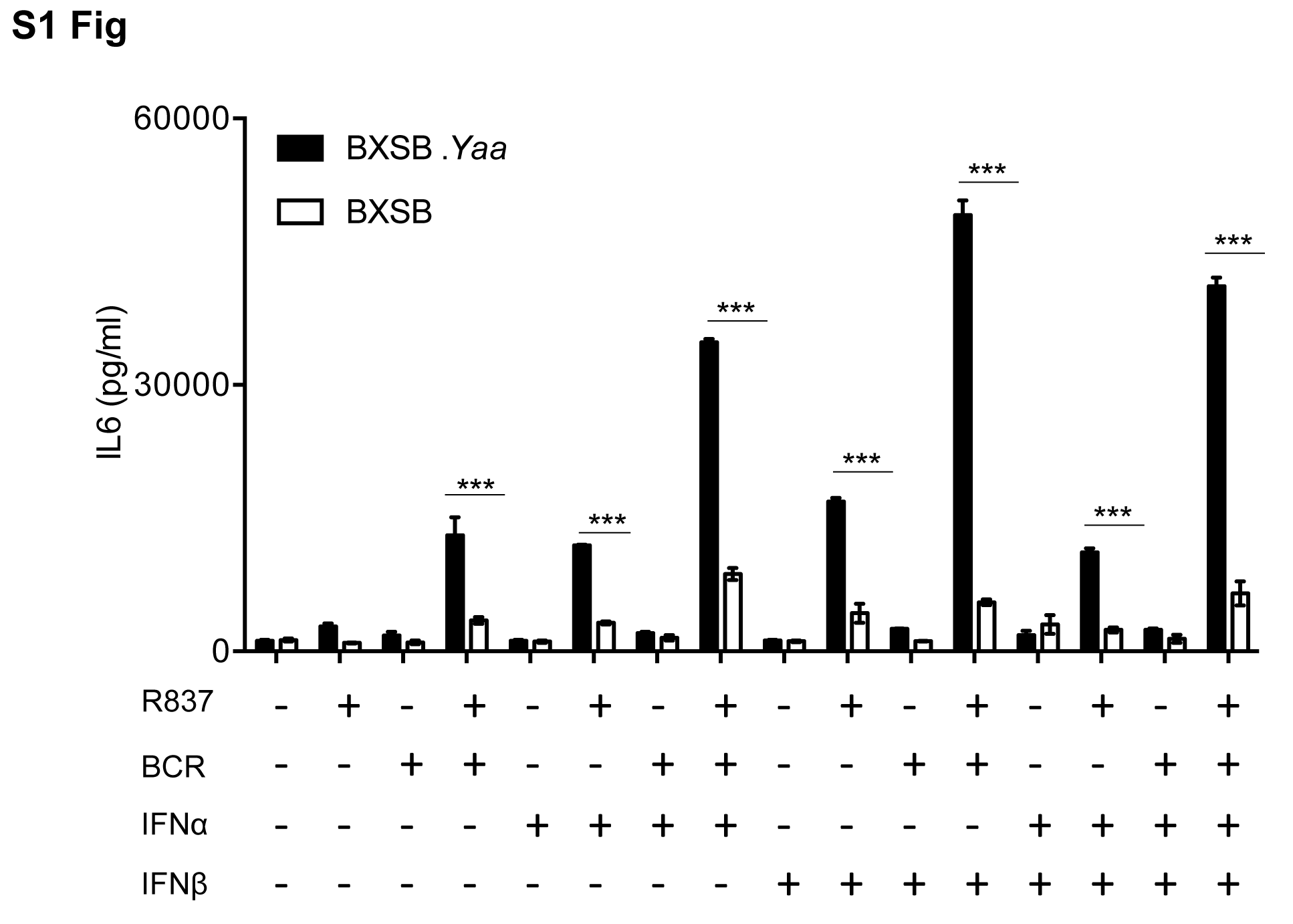

Supplement: S1 Fig — Purified B cells from BXSB.Yaa and BXSB mice were cultured in the presence or absence of R837 (50ng/ml); anti-BCR antibody (2g/ml); IFN and (40U/ml), either alone or in combinations, for 24h. Supernatants were collected and quantified for IL6 levels by standard sandwich ELISA method. Data is expressed as mean ± SEM of triplicate wells and is representative of two independent experiments. (TIF) [file pone.0153059.s001.tif]

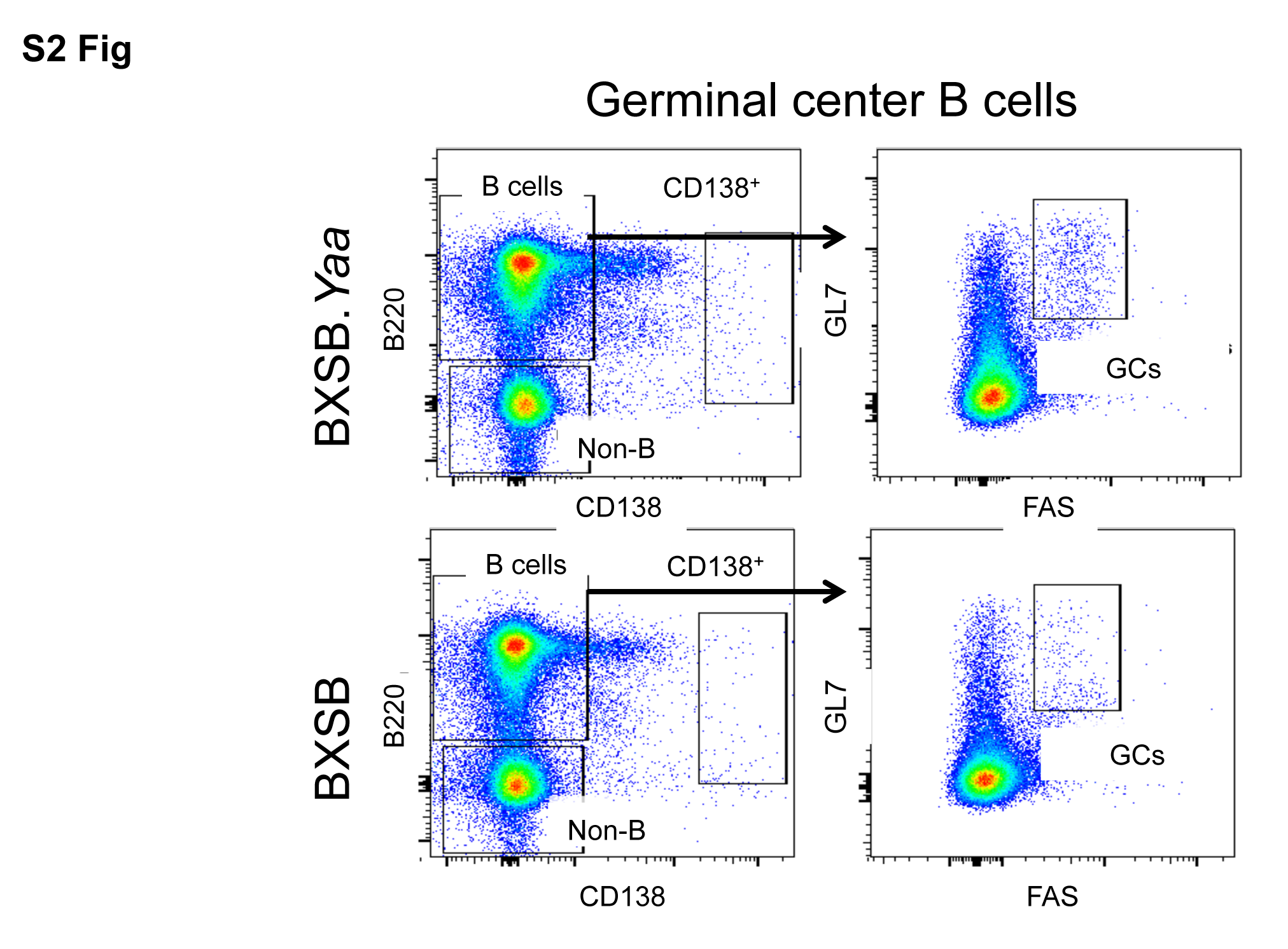

Supplement: S2 Fig — Splenocytes from BXSB.Yaa, BXSB.Yaa and BXSB mice were isolated and stained with anti-mouse antibodies to identify germinal center B cells (B220+GL7+Fas+). (TIF) [file pone.0153059.s002.tif]

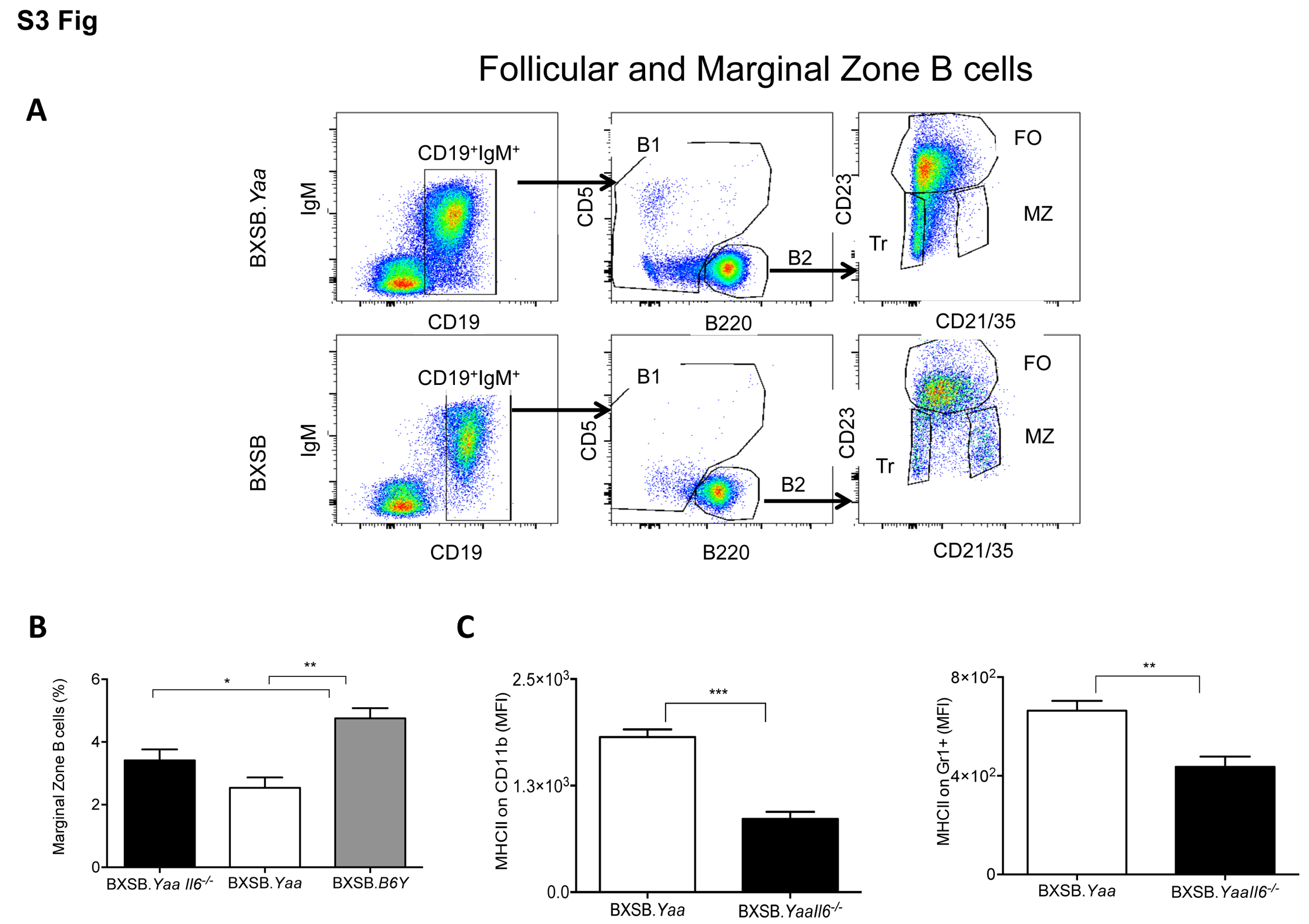

Supplement: S3 Fig — Splenocytes from BXSB.Yaa.Il6-/-, BXSB.Yaa and consomic BXSB.B6Y mice were isolated and stained with anti-mouse antibodies to determine (A-B) marginal zone B cells (CD19+IgM+B220+CD5-CD23-CD21+) and (B) MHCII expression on CD11b+ and Gr-1+ cells. (TIF) [file pone.0153059.s003.tif]

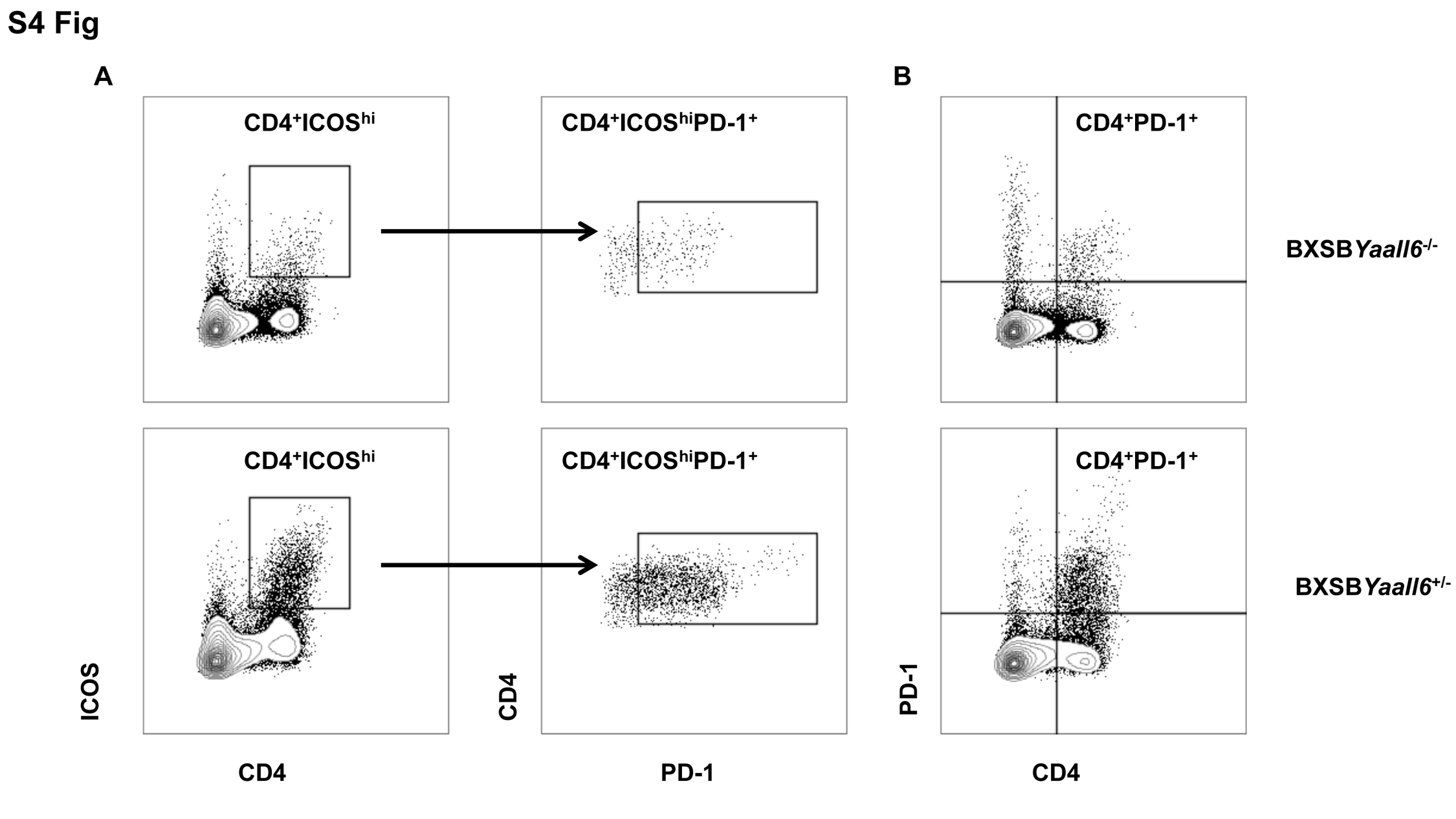

Supplement: S4 Fig — Splenocytes from B6.Yaa.Il6-/- and B6.Yaa Il6+/- mice were isolated and stained with anti-mouse antibodies to determine (A) CD4TFH cells (PD1+ ICOShi CD4+) and (B) PD1+ CD4+ cells. (TIF) [file pone.0153059.s004.tif]

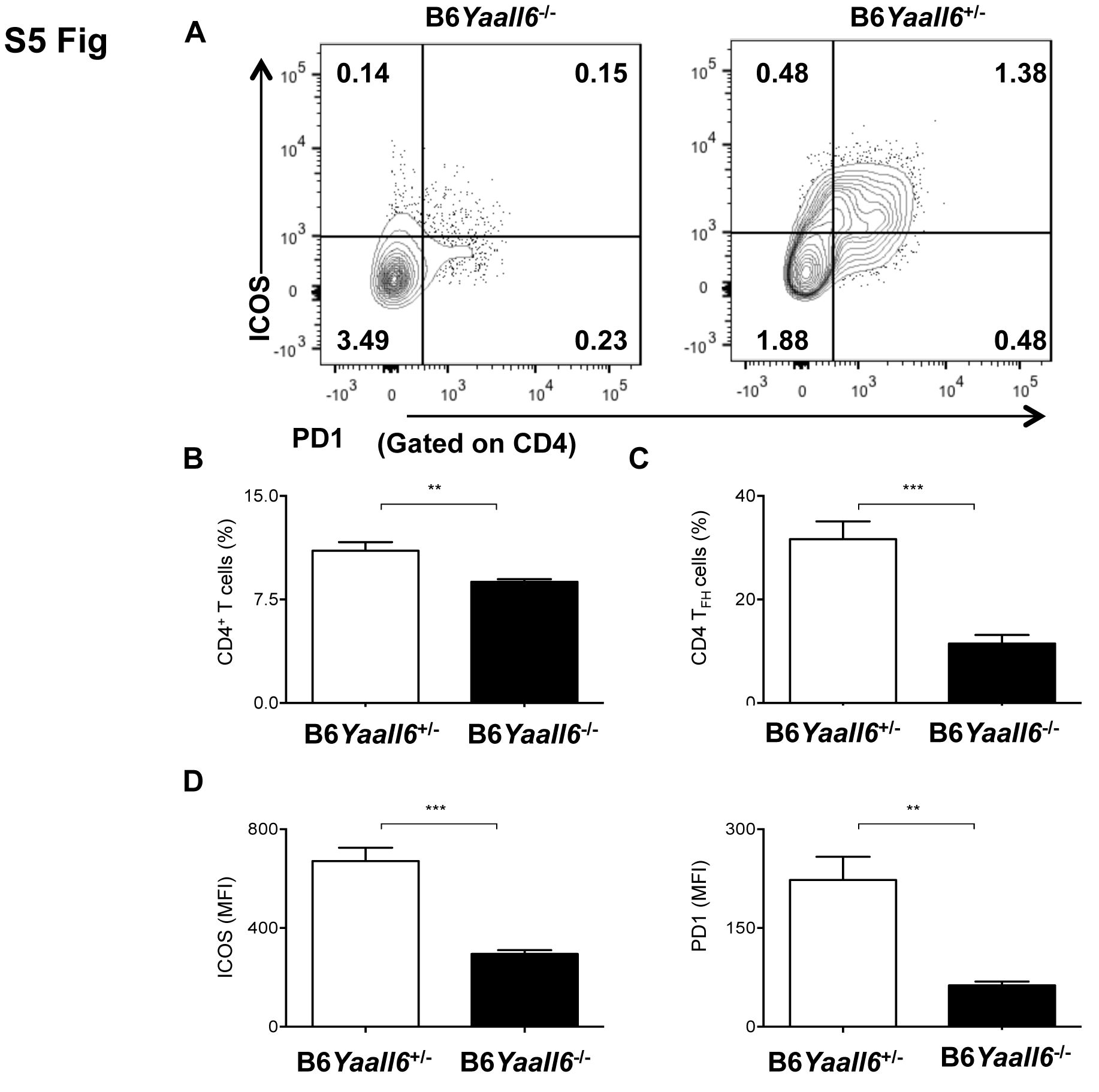

Supplement: S5 Fig — Splenocytes from B6.Yaa.Il6-/- and B6.Yaa Il6+/- mice were isolated and stained with anti-mouse antibodies. (A) FACS plots determine ICOS vs PD-1 expression on CD4+ T cells. Numbers in the plots represent percentage of total cells. Bar diagrams represent (B) percentages of CD4+ T cells (C) percentages of CD4TFH (PD1+ ICOS+ CD4+) cells (D) ICOS and PD-1 expression (MFI) on CD4 T cells. (B) and (C) represent frequencies of parent population. Data is mean ± SEM of 7–8 mice per group. P values were determined by two way ANOVA. (TIF) [file pone.0153059.s005.tif]
