# Supplementary material for: Interleukin 6 Accelerates Mortality by Promoting the Progression of the Systemic Lupus Erythematosus-Like Disease of BXSB.Yaa Mice
Source: PLoS One. 2016 Apr 6;11(4):e0153059. doi: 10.1371/journal.pone.0153059 (PMC4822786; doi:10.1371/journal.pone.0153059)
Supplement: S1 Table — (DOCX) [file pone.0153059.s006.docx]

# SUPPORTING INFORMATION

# Interleukin 6 Accelerates Mortality by Promoting the Progression of the Systemic Lupus Erythematosus-like Disease of BXSB.*Yaa* Mice

Shweta Jain^1^, Giljun Park^2,3^, Thomas J. Sproule^2^, Gregory J. Christianson^2^, Caroline M. Leeth^4^, Hongsheng Wang^1^, Derry C. Roopenian^2 *^, Herbert C. Morse III^1 *^

**S1 Table: List of Antibodies used in FACS and ELISA**

| ANTIBODY | SOURCE | FACS/ELISA | CLONE | FORMAT |
| --- | --- | --- | --- | --- |
| CD40 | BD | FACS | 3/23, 1C10 | FITC, PerCpefluor 710 |
| MHC-II | BD | FACS | AF6-120.1 | Pacific Blue |
| ICOSL | Biolegend | FACS | HK5.3 | Biotin |
| CD86 | BD | FACS | GL-1 | PECy5 |
| B220 | Biologend | FACS | RA3-6B2 | APCCy7, Alexa 700, Pacific Orange |
| IgM | Biolegend | FACS | RMM-1 | PECy7 |
| IL21R | BD | FACS | 4A9 | PE |
| FAS | BD | FACS | DX2 | PE |
| CD19 | Biolegend | FACS | 6D5 | APCCy7, Alexa 700, Pacific Blue, APC |
| CD5 | Biolegend | FACS | 53-7.3 | PECy5 |
| CD23 | BD | FACS | B3B4 | PE |
| CD21 | BD | FACS | 7E9 | FITC, APCCy7 |
| PNA | Sigma | FACS | NA | Biotin |
| GL7 | BD | FACS | GL7 | FITC |
| CD4 | Biolegend | FACS | GK1.5 | APCCy7, Alexa 700, Pacific Blue, APC, FITC |
| ICOS | BD | FACS | C398.4A | PECy5 |
| PD-1 | BD | FACS | 29F.1A12 | APC |
| CD11b | Biologend | FACS | M1/70 | PECy7 |
| CD11c | Biologend | FACS | N418 | PE |
| mPDCA | BD | FACS | eBio927 | PerCpefluor 710 |
| Gr-1 | BD | FACS | RB6-8C5 | FITC, APC |
| CXCR5 | BD | FACS | L138D7 | PE, Pacific Blue, PercpCy5.5 |
| IL6 | BD | ELISA | MP5-20F3 | Purified |
| IL6 | BD | ELISA | MP5-32C11 | Biotin |
| IgG2b | BD | ELISA | R9-91 | Purified |
| IgG2b | BD | ELISA | R12-3 | Biotin |
| IL21 | Peprotech | ELISA | Polyclonal Rabbit | Purified |
| IL21 | Peprotech | ELISA | Polyclonal Rabbit | Biotin |

## Supplemental Figure Legends

**S1 Fig. Concomitant signaling through TLR, BCR and type I interferon enhances IL6 secretion by BXSB.*Yaa* B cells.** Purified B cells from BXSB.*Yaa* and BXSB mice were cultured in the presence or absence of R837 (50ng/ml); anti-BCR antibody (2μg/ml); IFNα and β (40U/ml), either alone or in combinations, for 24h. Supernatants were collected and quantified for IL6 levels by standard sandwich ELISA method. Data is expressed as mean ± SEM of triplicate wells and is representative of two independent experiments.

**S2 Fig. Gating strategy for germinal center B cells.** Splenocytes from BXSB.*Yaa*, BXSB.*Yaa* and BXSB mice were isolated and stained with anti-mouse antibodies to identify germinal center B cells (B220^+^GL7^+^Fas^+^)

**S3 Fig. Abrogating IL6 signaling diminishes marginal zone B cell frequencies and activation of monocytes.** Splenocytes from BXSB.*Yaa.Il6*^-/-^, BXSB.*Yaa* and consomic BXSB.*B6Y* mice were isolated and stained with anti-mouse antibodies to determine (A-B) marginal zone B cells (CD19^+^IgM^+^B220^+^CD5^-^CD23^-^CD21^+^) and (B) MHCII expression on CD11b^+^ and Gr-1^+^ cells.

**S4 Fig. Gating strategy for CD4 T_FH_ cells.** Splenocytes from B6.*Yaa.Il6*^-/-^ and B6.*Yaa Il6*^+/-^ mice were isolated and stained with anti-mouse antibodies to determine (A) CD4T_FH_ cells (PD1^+^ ICOS^hi^ CD4^+^) and (B) PD1^+^ CD4^+^ cells.

**S5 Fig.** **IL6 acts upstream of IL21 in the disease progression of BXSB.*Yaa* mice.** Splenocytes from B6.*Yaa.Il6*^-/-^ and B6.*Yaa Il6*^+/-^ mice were isolated and stained with anti-mouse antibodies. (A) FACS plots determine ICOS vs PD-1 expression on CD4^+^ T cells. Numbers in the plots represent percentage of total cells. Bar diagrams represent (B) percentages of CD4^+^ T cells (C) percentages of CD4T_FH_ (PD1^+^ ICOS+ CD4^+^) cells (D) ICOS and PD-1 expression (MFI) on CD4 T cells. (B) and (C) represent frequencies of parent population. Data is mean ± SEM of 7-8 mice per group. P values were determined by two way ANOVA.
